# Participants in *Treponema pallidum* pathogenesis: progress in functional proteins

**DOI:** 10.3389/fimmu.2025.1632677

**Published:** 2025-08-26

**Authors:** Wei Zuo, Yongjian Xiao, Qing Xiang, Shuangwen Xiao, Yafeng Xie

**Affiliations:** Department of Clinical Laboratory, The Second Affiliated Hospital, Hengyang Medical School, University of South China, Hengyang, China

**Keywords:** *Treponema pallidum*, functional protein, interaction, pathogenesis, immune evasion

## Abstract

*Treponema pallidum subsp. pallidum (T. pallidum)* is the causative agent of syphilis, a chronic sexually transmitted disease that leads to widespread organ damage. The pathogenesis of syphilis involves crucial functional proteins that facilitate bacterial adhesion to host cells, invasion, dissemination, immune evasion, and inflammatory responses. Investigating these proteins is crucial for the development of innovative diagnostic tools, vaccines, and therapies. However, the intricate nature of *T. pallidum* and the inability to culture *in vitro* hinder our comprehensive understanding of these proteins. This review article presents innovative understandings of the pathogenesis of *T. pallidum* functional proteins, building upon existing knowledge. This paper establishes a foundation for comprehending the current knowledge landscape and outlining future research avenues.

## 1 Introduction

Syphilis, a chronic sexually transmitted disease caused by *Treponema pallidum subsp. pallidum* (*T. pallidum*), poses a significant threat to human health due to its potential for multi-organ damage (1). Syphilis treatment and clinical management must adhere to the principles of “early diagnosis, prompt treatment, and adequate standardized medication.” Penicillin-based regimens are the first-line therapy, adjusted according to disease stage and individual circumstances. This approach emphasizes synchronized partner screening and treatment, along with rigorous serological monitoring (early and sufficient penicillin treatment can achieve cure). Maternal and neonatal management constitutes a critical component for blocking mother-to-child transmission (2). Global efforts are intensifying screening and expanding accessible services to curb the epidemic. According to the latest data from the Global Burden of Disease Study 2021, there were approximately 18.696 million incident cases of syphilis globally in 2021, with an age-standardized incidence rate of 235.47 per 100,000 population (3). Recent epidemiological data in China indicate a notable rise in syphilis incidence (4). However, the inability to cultivate and genetically manipulate *T. pallidum* in the laboratory has impeded the elucidation of its molecular pathogenic mechanisms. *T. pallidum*, a member of the genus Treponema within the family Treponema, features a structure comprising a cylindrical protoplasm, a flagella-like structure, a plasma membrane, a peptidoglycan layer, a periplasmic space, and an outer membrane arranged from inner to outer layers (5). Notably, the outer membrane lacks lipopolysaccharides typical of Gram-negative bacteria and does not secrete lytic or cytotoxic toxins; nevertheless, it exhibits robust tissue invasion capabilities. During the initial stages of infection, *T. pallidum* can breach the placental and blood-brain barriers, leading to congenital syphilis and neurosyphilis, respectively (6). Previous investigations have outlined the pathogenic cascade of *T. pallidum*, encompassing adhesion, invasion, immune evasion, dissemination, and tissue damage. Numerous studies have delved into the functional proteins’ roles in *T. pallidum* pathogenesis. This review will summarize advancements in understanding the pathogenic mechanisms of the agent’s functional proteins across these processes (**Table 1**).

**Table 1:** Key functional proteins of *Treponema pallidum* and their pathogenic mechanisms.

Protein name	Molecular characteristics	Binding receptors/targets	Main pathogenic mechanisms
Tp0751	75 kDa, contains β-sheet domains and HEXXH metalloprotease motif, C-terminal β-barrel structure (9)	Laminin receptor (LamR), Fibrinogen (FG)	Mediates adhesion (13), penetration, basement membrane degradation (38), inflammatory activation (11).
Tp0136	50 kDa, contains N-terminal fibronectin-binding domain (FNBD), C-terminal lipidation	Fibronectin (FN), Vitronectin (VN)	Tissue colonization (19), host cell internalization, antigen masking.
Tp0954	Lipoprotein, contains heparan sulfate-binding domain (HSBD)	Heparan sulfate (HS), Dermatan sulfate (DS)	Placental penetration (22), tissue dissemination, immune suppression.
Tp0435(Tp17)	14 kDa (54), contains antiparallel β-barrel structure, can form disulfide-linked dimers (56)	Host cell surface glycosaminoglycans (GAGs)	antigen masking (55), persistent adhesion (neural/placental).
TprK(Tp0897)	Variable antigen (V1-V7 regions) (66), generates diversity via gene conversion	Host antibodies, T-cell receptors (TCR)	Antigen variation (51), T-cell exhaustion, latency extension.
Tp0821	26.8 kDa, outer membrane lipoprotein, contains TLR2/CD14-binding domain	TLR2, CD14	Inflammatory activation (72), endothelial damage, immune imbalance (pro-inflammatory factor upregulation) (26).

## 2 Adhesion and colonization

*T. pallidum*, as a microaerophilic bacterium, is compelled to adhere to and colonize host cells due to its severe biosynthetic deficiencies and ineffective reactive oxygen species (ROS) scavenging mechanisms. This strategy serves dual purposes: direct nutrient acquisition and dependence on host antioxidant systems (e.g., catalase) to mitigate oxidative stress for survival. By employing specific adhesion facilitated by functional proteins, *T. pallidum* establishes targeted attachment to mucopolysaccharide-rich tissues such as the skin and aorta (7). This adhesion and colonization process represents the initial stages of *T. pallidum* infection, where surface proteins of *T. pallidum* interact with the host extracellular matrix (ECM) and receptors through various mechanisms, setting the stage for subsequent invasion.

### 2.1 Tp0751

Tp0751 comprises an N-terminal disordered region (IDR) and a C-terminal lipocalin fold (8). The C-terminal domain is composed of eight beta chains that create an unconventional lipid vesicle protein fold. This domain features multiple short conserved regions scattered on its surface, establishing an interface with extracellular matrix (ECM) components (9). Moreover, the C-terminus of this domain harbors a zinc ion-catalyzed HEXXH metalloprotease activity motif responsible for degrading host proteins like fibrinogen and laminin (10). Recent studies further reveal that Tp0751 contributes to blood-brain barrier disruption by altering tight junction protein expression (e.g., ZO-1, occludin) and inducing pro-inflammatory cytokines (e.g., IL-6) in endothelial cells (11). Tp0751 is situated external to the plasma membrane of *T. pallidum*, rather than on the outer membrane surface, leading to its limited immunogenicity. Heterogeneous expression studies demonstrate its capacity to mimic adhesion functions on the surface of *Borrelia burgdorferi*, affirming its potential for surface exposure (12). The adhesion mechanism of the Tp0751 protein involves utilizing multiple domains to collectively recognize host extracellular matrix (ECM) components such as laminin, fibronectin. It facilitates electrostatic binding through arginine/lysine-rich regions and generates a multivalent anchoring effect (13). Critically, Tp0751 disrupts endothelial integrity by targeting VE-cadherin junctions, thereby facilitating bacterial transmigration across endothelial barriers (14). Under shear stress conditions, Tp0751 facilitates adhesion through a two-stage mechanism: an initial deceleration stage, wherein it extensively binds to extracellular matrix (ECM) components, thereby reducing helicoid movement speed; and a subsequent specific binding stage, during which it interacts with endothelial receptors (e.g., ICAM-1) via the p10 region to establish stable adhesion. Experimental evidence has demonstrated that synthetic peptides derived from the lipocalcin domain of Tp0751 can impede its adhesion capacity (9). Vaccination studies in rabbits demonstrate that antibody-mediated blockade of Tp0751’s adhesive functions significantly inhibits T. pallidum dissemination, highlighting its pivotal role in systemic spread without invoking thrombin degradation (15). Tp0751 has been demonstrated to bind multiple extracellular matrix (ECM) proteins. The aberrant deposition of these proteins within the tumor microenvironment provides a foundation for its targeting potential (16). Future research should explore Tp0751-functionalized nanoparticles to enhance tumor-targeted delivery efficiency through specific ECM binding. Integrating imaging probes with therapeutic payloads could facilitate the development of theranostic nanoplatforms.

### 2.2 Tp0136

Tp0136, a 495-amino-acid protein with a molecular weight of approximately 50 kDa, is encoded by a 1,488 bps gene. Tp0136 exhibits significant sequence heterogeneity among different strains (17). This manifests as nucleotide mismatches, insertions, or deletions in the coding gene, leading to amino acid sequence diversity, and such differences remain stable across different isolates (18). In the pathogenesis of *T. pallidum* adhesion, the Tp0136 protein utilizes a complex molecular mechanism. It selectively binds to host extracellular matrix (ECM) components in the conserved N-terminal region and variable C-terminal regions (18). Particularly, its binding affinity for cellular fibronectin (cFn) exceeds that for plasma fibronectin (pFn), highlighting its essential function in tissue penetration (18). In human dermal vascular smooth muscle cells (HDVSMCs), Cai et al. demonstrated that recombinant Tp0136 protein activates the PI3K, MAPK (JNK, p38), and NF-κB signaling pathways. This activation led to a significant, concentration-dependent upregulation of matrix metalloproteinase-1 (MMP1) at both mRNA and protein levels, while TIMP1 and TIMP2 expression remained largely unchanged. Consequently, the MMP1/TIMP1 and MMP1/TIMP2 ratios were significantly increased. Pharmacological inhibition of these pathways suppressed MMP1 induction and restored the MMP/TIMP balance. These findings suggest that Tp0136 promotes ECM degradation and facilitates T. pallidum dissemination by disrupting the MMP/TIMP equilibrium via PI3K/MAPK/NF-κB signaling in vascular smooth muscle cells (19). Djokic et al. (20) observed that when *Borrelia burgdorferi* expressed Tp0136 heterologously (B314 strain), it displayed varying affinities for different host cell lines, with the highest affinity observed for epidermal cells (HEK293) and glial cells (C6). Furthermore, the protein disrupts vascular endothelial cell-to-cell junctions, including VE-cadherin, in a dose-dependent manner. This disruption results in a threefold increase in endothelial permeability above baseline levels within 24 hours, promoting spirochete hematogenous dissemination (21). The mechanisms mentioned above work together to anchor pathogens to infection sites and facilitate systemic dissemination by altering host microenvironments, providing essential molecular targets for targeted intervention strategies to inhibit pathogen adhesion.

### 2.3 Tp0954

Tp0954, a surface lipoprotein of *T. pallidum*, plays a pivotal role as a placenta-targeted adhesin (22). Its pathogenic role is associated with its tetratricopeptide repeat (TPR) domain, which mediates specific interactions with host tissues and forms oligomers, significantly enhancing the pathogen’s adherence to host tissues, particularly glycosaminoglycans like dermatan sulfate, heparin, and heparan sulfate (23). This interaction facilitates the binding of spirochetes to placental trophoblast cells. In experimental models, the adhesion efficiency of Tp0954 increases by more than 50%. This targeted adhesion not only assists in the traversal of the pathogen through the placental barrier but also promotes vertical transmission from mother to child by disrupting intercellular junction structures, a fundamental mechanism in the pathogenesis of congenital syphilis (22). He et al. demonstrated through heterologous expression of Tp0954 that this protein significantly enhances adhesion to epithelial, endothelial, neuronal, and placental cells in heterologous hosts such as the *Borrelia burgdorferi* B314 strain (22, 24). Their findings indicate that Tp0954 mediates *T. pallidum* adhesion to host cells, thereby promoting initial pathogen colonization and transplacental transmission. Future studies should focus on elucidating the specific interaction mechanisms between Tp0954 and host cell surface receptors, as well as exploring how its adhesion functionality regulates immune evasion and systemic dissemination. *In vitro* studies have shown that overexpression of Tp0954 significantly enhances spirochete colonization in placental tissues, highlighting its potential as a critical target for preventing mother-to-child transmission.

### 2.4 Tp0155

Tp0155, consisting of 371 amino acids and has a molecular weight of approximately 43 kDa, is cell surface localized (25). It plays a crucial role in the adhesion process of *T. pallidum* due to its distinctive domain structure and dynamic expression profile. The protein facilitates the pathogenicity of *T. pallidum* by utilizing two key domains, namely LysM and M23. The LysM domain specifically interacts with fibronectin (FN) present on the surface of host cells. The M23 domain acts as a peptidase that degrades peptidoglycans, thereby facilitating tissue invasion (26). Similarly, studies suggest that the M23 domain in Treponema denticola may degrade extracellular matrix components to support bacterial colonization. Tp0155 collaborates with Tp0483 to establish an adhesion system where Tp0155 anchors matrix FN for direct adhesion, while Tp0483 facilitates indirect binding of soluble FN (27). Throughout the infection process, Tp0155 demonstrates a dual-phase expression pattern during the chancre and immune clearance phases. It maintains high expression levels to evade host defenses effectively. Moreover, its coordinated action with the metalloproteinase Tp0751, which enhances adhesion and degrades the matrix, accelerates the dissemination of the pathogen. Targeting the LysM domain with small-molecule inhibitors presents a promising approach to impede FN binding, offering a novel avenue for anti-adhesion therapy.

## 3 Invasion and dissemination

*T. pallidum* exhibits a robust capacity for invasion and infection, leading to various clinical presentations throughout the course of syphilis, which is characterized by three stages: primary syphilitic chancre, secondary syphilis with mucocutaneous lesions, and tertiary syphilis involving gummatous lesions and cardiovascular/neurological complications (28). This pathogen can disseminate systemically via blood and lymphatic routes, breaching the blood-brain, blood-testis, and placental barriers (29). Upon successful colonization, the pathogen employs a sophisticated “molecular drilling system” comprising multiple protein modules to facilitate systemic dissemination by simultaneously dismantling physical barriers and modulating the immune microenvironment.

### 3.1 Tp0965

Tp0965 protein, with a molecular weight of approximately 35.5 kDa, is a periplasmic membrane fusion protein encoded by the tp34 gene cluster (30). Its robust immunogenicity and interactions with host cells are pivotal in the pathogenesis process (31). Generally, this protein can facilitate pathogen dissemination by disrupting vascular barriers through a multifaceted mechanism. Specifically, Tp0965 activates endothelial cells, leading to a substantial upregulation of adhesion molecules such as ICAM-1, E-selectin, and the chemokine MCP-1 (30). Moreover, it enhances monocyte mobility by 34.8%. Additionally, Tp0965 induces F-actin reorganization in endothelial cells, resulting in the formation of intracellular stress fibers and a 50% reduction in the expression of the tight junction protein Claudin-1. Consequently, vascular permeability is significantly increased, as evidenced by a 130% rise in HRP flux, thereby expediting pathogen infiltration into deeper tissues. Studies on the molecular mechanisms have revealed that Tp0965 facilitates cytoskeletal remodeling and barrier damage via the RhoA/ROCK pathway, with partial reversal of its effects by a ROCK inhibitor (30). Furthermore, it triggers the activation of the ERK/JNK/p38 MAPK signaling pathway depending on the TLR2/Chemerin-ChemR23 axis (32). This leads to the upregulation of Cemerin-dependent inflammatory factors, such as MMP-2, thereby exacerbating endothelial damage and immunopathological effects. The structural characteristics of Tp0965, including its membrane fusion properties and regulation of signaling cascades, collectively disturb vascular barrier homeostasis. As a highly sensitive antigen and a critical signaling target (e.g., RhoA/ROCK), Tp0965 serves as a crucial molecular determinant for understanding the mechanisms underlying syphilitic vascular injury and for devising targeted intervention strategies (30).

### 3.2 TpF1

TpF1, a 17.2 kDa monomer forming a 206 kDa dodecamer (33), shares structural homology with *Helicobacter pylori* HP-NAP and exhibits pro-inflammatory properties (34). It serves as an early serological marker, though diagnostic sensitivity data requires validation (35). In human umbilical vein endothelial cells (HUVECs), TpF1 activates the CREB/NF-κB signaling, elevating IL-8 expression 3-fold. Consequently, endothelial proliferation, migration, and microangiogenesis are significantly enhanced in an IL-8-dependent manner, as evidenced by 50% more vessel branches in zebrafish (34). This process is crucial in secondary syphilis, where IL-8-mediated neovascularization facilitates pathogen dissemination and neutrophil recruitment, exacerbating local inflammation. Conversely, TpF1 suppresses microglial migration by impairing actin polymerization via TLR4/PI3K/AKT/Rac1 inhibition, reducing F-actin/G-actin ratio and impairing cytoskeletal dynamics. This dual modulation of host responses—enhancing vascularization while disabling microglial motility—may facilitate systemic dissemination (36). Theoretical targeting of IL-8 pathways could attenuate angiogenic effects (34). The dual functionality of TpF1 as both a diagnostic marker and a pathogenic factor, along with its conserved structural characteristics, lays the groundwork for the development of innovative diagnostic and therapeutic approaches.

### 3.3 Tp0751 (pallilysin)

Tp0751 is a bifunctional outer membrane protein with the capacity to bind laminin (LN), fibronectin (FN), and fibrinogen (FG), which are present on endothelial cell surfaces and in the underlying basement membrane (37). Additionally, it exhibits metalloproteinase activity, known as pallilysin, enabling the degradation of bound laminin and fibrinogen (38). Structurally, the C-terminal region of Tp0751 features a predicted beta-barrel structure composed of eight beta-strands, belonging to the OmpA-OmpF porin-like superfamily (9). This region harbors the HEXXH metalloproteinase motif, facilitating Zn²^+^ binding and functioning as a zinc-dependent membrane-bound metalloproteinase (10). Studies by Houston et al. (38) demonstrated that mutations in the HEXXH motif do not impact Tp0751’s adhesion function, suggesting a structural and functional independence between its adhesion and protease activities. The proteolytic action of pallilysin on fibrin clots promotes the dissemination of *T. pallidum* within the host, aiding in the establishment of chronic infection. Recent work by Lithgow et al. (14) revealed that both live *T. pallidum* and recombinant Tp0751 can disrupt VE-cadherin without altering vascular barrier permeability. This finding, along with supporting data on cholesterol dependence, led Lithgow et al. to propose a model where *T. pallidum* traverses the endothelial barrier via cholesterol-dependent endocytosis.

### 3.4 Tp0750

Tp0750, a serine protease located at out membrane in *T. pallidum*, functions in adhesion. The gene encoding it spans 672 bp, translating into a 223-amino-acid protein with a molecular weight of approximately 26 × 10³. Structurally and functionally akin to Tp0751, Tp0750 serves as a pivotal virulence factor in *T. pallidum*, facilitating host barrier penetration and systemic dissemination. Noteworthy features of this protein include vWFA domains and metal ion-dependent adhesion sites (MIDAS), enabling interactions with host proteins like annexin A2 through calcium ion binding (39). Moreover, Tp0750 modulates the coagulation-fibrinolytic system by exhibiting dual enzymatic properties (serine protease and metalloproteinase-like activities). Functionally, Tp0750 impedes thrombosis by degrading fibrinogen (Fg) and fibronectin (Fn), while its interaction with annexin A2 promotes plasminogen activation, hastening pathogen release. Additionally, its metalloproteinase-like activity collaborates with pallilysin (Tp0751) to degrade ECM components like laminin and collagen, instigating a sequence of events termed “basement membrane degradation-deep invasion” (39). Moreover, Tp0750 orchestrates an intricate “invasion-lysis” cycle by modulating endothelial hemostasis equilibrium, suppressing anticoagulant factors, and stimulating the secretion of pro-inflammatory factors, which significantly enhances vascular permeability, thereby facilitating the penetration of spirochetes through endothelial barriers. The concurrent transcription and functional synergy with pallilysin’s genome, involving enzyme activity division and collaboration in thrombus inhibition, further intensifies the destructive impact on tissues. The regulation of targeting through its structural configuration and the coordination within a multi-enzyme network establishes the molecular underpinning for the widespread dissemination of *T. pallidum*. Additionally, in the view of its highly conserved pivotal functional domains, Tp0750 may serve as a promising candidate for vaccine development (40).

## 4 Immune evasion

*T. pallidum* has exhibited robust immune evasion capabilities, capacitating it to withstand host immune responses, with functional proteins playing a pivotal role in this phenomenon. Specifically, the pathogen evades individual immune defense and elimination through the generation of numerous antigenic variations. Additionally, certain functional proteins impede the activation and replication of host immune cells such as T cells, B cells, and macrophages, facilitating the establishment of prolonged latency and sustaining transmission (41) (**Table 2**).

**Table 2:** Immune evasion mechanisms and key proteins.

Protein name	Immune evasion mechanism	Associated pathways/molecules
TprK	Antigenic variation through gene conversion to escape immune recognition (66); suppression of T cell function to prolong latency (53).	Gene conversion mechanism, T cell receptor (TCR)
Tp0326 (Tp92)	Induction of pyroptosis and apoptosis in monocytes (48); inhibition of neutrophil apoptosis (47); regulation of IL-8 secretion to interfere with immune clearance (48).	TLR2/CD14, RIPK1/caspase-8/caspase-3 (48)
Tp0435 (Tp17)	Random surface/periplasmic expression (55); low immunogenicity with no antibody response in 70% of patients, escaping humoral immunity.	Host glycosaminoglycans (GAGs)
Tp0574 (Tp47)	Inhibition of macrophage phagocytic function; induction of PGE2 via the PERK/NF-κB/COX-2 pathway to weaken host defense (59).	PI3K/AKT/mTOR, autophagy pathway

### 4.1 Tp0326 (Tp92)

Tp0326 (Tp92) is the sole outer membrane protein (OMP) within the *T. pallidum* family that shares homology with Escherichia coli’s BamA (42). It is the singular protein in the genome exhibiting sequence similarity to OMPs found in known Gram-negative bacteria (43). Belonging to the Omp85 superfamily, Tp0326 features a β-barrel structure and is characterized by low expression levels (44). Its structural attributes encompass the N-terminal POTRA domain, which facilitates outer membrane protein folding, and the C-terminal 18-strand β-barrel transmembrane structure. This structure exposes functional epitopes on the cell surface, albeit its limited abundance (approximately 100 copies/cell) and variations in the conformation of recombinant proteins hinder host immune recognition (45). Notably, It reveals that *T. pallidum* evades immune attacks by restricting the immunogenicity of surface antigens (such as β-barrels) and preferentially exposing non-surface regions (such as POTRA domains) (43). Furthermore, Tp92 not only contributes to immune evasion by restricting the immunogenicity of surface epitopes, but also actively promotes inflammation; for instance, it has been shown to activate endothelial cells and induce the expression of pro-inflammatory mediators. Tp92 induces apoptosis in monocytic cells (THP-1) through the RIPK1/caspase-8/caspase-3 pathway, promotes IL-8 secretion, resulting in depletion of immune cells (46). Conversely, it suppresses apoptosis in human polymorphonuclear neutrophils (hPMNs) via activation of ERK MAPK, PI3K/Akt, and NF-κB signaling pathways, thereby prolonging neutrophil survival (47). This anti-apoptotic effect is mediated by upregulation of the anti-apoptotic protein Mcl-1, inhibition of caspase-3/8/9 activity, and maintenance of mitochondrial membrane potential. Furthermore, Tp92 activates endothelial cells through the chemerin/CMKLR1 axis, inducing TNF-α and ICAM-1 expression to promote macrophage migration (48). The imbalanced “pro-inflammatory-anti-apoptotic” strategy exacerbates vascular permeability and tissue damage by inducing excessive IL-8 levels. Moreover, it creates a conducive environment for pathogen dissemination across physiological barriers like the blood-brain barrier by depleting immune cell populations.

### 4.2 TprK (Tp0897)

TprK (Tp0897) serves as the primary immune evasion factor in *T. pallidum (* 49). Its structure comprises 20 β-barrel pore proteins with 10 surface-exposed loops, including 7 variable regions (V1-V7) that undergo sequence diversification via gene conversion mechanisms (50). Meanwhile, conserved regions (C1-C3) ensure structural stability. This dynamic variability enables host antibodies to target variable epitopes within the V region, promoting immune evasion. For instance, the SS14-DCKO strain exhibits reduced virulence *in vivo* due to its inability to generate new variants, underscoring the importance of antigenic diversity for pathogen survival (51). Pre-existing antibodies, such as anti-V6 antibodies, can drive specific sequence remodeling in corresponding regions, initiating an “immune selection-escape” cycle (52). Furthermore, the segregation of B and T cell epitopes in TprK (with B cell epitopes predominantly in variable regions and T cell epitopes primarily in conserved regions) hinders effective immune clearance (53). Although its outer membrane transport function is dispensable for *in vitro* growth, it serves as a virulence factor under immune pressure *in vivo*. The substantial variability of TprK presents significant obstacles to vaccine development; however, approaches that focus on conserved regions or utilize multivalent antigens have the potential to overcome protective constraints, offering novel avenues for managing the chronicity and transmission of syphilis.

### 4.3 Tp0435 (Tp17)

Tp0435 (Tp17), a 14 kDa periplasmic lipoprotein antigen of *Treponema pallidum* encoded by one of the pathogen’s most highly expressed genes (54), is characterized by an antiparallel beta-barrel structure comprising eight beta chains (55). Unlike conventional transmembrane proteins, Tp0435 lacks a central channel and is filled with amino acid side chains, suggesting a distinct function in mediating adhesion through membrane organization or ligand binding (55). Regarding immune evasion mechanisms, it undergoes lipidation (e.g., palmitoylation) generating multiple isoforms, as observed in heterologous expression systems like *Borrelia burgdorferi* (56). These isomers are partially exposed on the surface while mostly concealed in the periplasmic space, effectively evading antibody recognition. Keane et al. reported antibody reactivity to dominant Tp0435 epitopes (peptides 1,4,5) in 95-100% of primary/secondary syphilis sera versus 67-83% in late latent stage, indicating temporal downregulation to evade adaptive immunity (57). Subsequently, its expression is down-regulated to evade adaptive immunity, thereby supporting the long-term latency of spirochetes in immune-privileged sites such as the central nervous system. Furthermore, the preferential colonization of immune-privileged tissues contributes to attenuating the systemic immune response of the host. These mechanisms demonstrate how Tp0435 contributes to the chronicity of syphilis by adapting structurally and evading the immune system, thus offering a fundamental molecular foundation for specific interventions.

### 4.4 Tp0574 (Tp47)

Tp47, a 367-amino acid hydrophobic outer membrane protein anchored via N-terminal lipidation, mediates immune evasion through multifaceted mechanisms. In macrophages, Tp47 triggers endoplasmic reticulum stress pathways (PERK/ATF4 and IRE1/XBP1), inducing autophagy and promoting prostaglandin E2 (PGE2) synthesis (58). PGE2 suppresses phagosome maturation via autocrine EP2 receptor signaling, impairing bacterial clearance—an effect reversible by COX-2 inhibitors (e.g., celecoxib) (59). Concurrently, Tp47 competitively binds to pyruvate kinase M2 (PKM2), inhibiting its ubiquitin-dependent degradation. This stabilizes phosphorylated PKM2-Y105, enhancing glycolytic flux (3-fold increase in lactate) and activating EIF2AK2 phosphorylation, which drives NLRP3 inflammasome assembly and caspase-1-mediated IL-1β release (60). The resulting inflammatory storm causes tissue damage and depletes anti-inflammatory resources (e.g., IL-1RA). For promoting leukocyte adhesion and endothelial activation, Tp47 stimulates monocytes to secrete MMP-2-enriched microvesicles (Tp47-MVs). These activate the ERK1/2-NF-κB cascade in endothelial cells, upregulating ICAM-1/VCAM-1 to promote monocyte transendothelial migration, while MMP-2 degrades tight junction proteins (occludin/claudin-5) (61). Additionally, in fibroblasts, Tp47 induces proinflammatory mediators via the PI3K/Akt-p38MAPK-NF-κB axis (62), recruiting monocytes and promoting leukocyte adhesion to promote inflammatory vasculopathy. Furthermore, by blocking PKM2 ubiquitination, Tp47 exacerbates lactate-driven EIF2AK2-NLRP3 activation, inducing pyroptotic inflammatory senescence in macrophages (60). Tp47 inhibits the mTOR pathway, provoking autophagy-dependent microglial death (63). Collectively, while its high immunogenicity establishes it as a core serodiagnostic marker (64), Tp47 orchestrates immune evasion by suppressing phagocytosis, inciting inflammatory cascades, disrupting vascular integrity, reprogramming immunometabolism, and facilitating neural invasion.

## 5 Inflammation induction

Following infection with *T. pallidum*, local tissue inflammation ensues, facilitated by the release of toxins and enzymes, including plasminogen activator and hyaluronidase, which directly harm the extracellular matrix and intercellular connections of host cells, leading to tissue structural deterioration and functional impairment. Moreover, the infection can trigger apoptosis and necrosis of host cells, exacerbating tissue injury. The pathogenic proteins of *T. pallidum* contribute to tissue damage and the persistence of infection by orchestrating a delicate interplay between pro-inflammatory and anti-inflammatory signaling pathways (**Table 3**).

**Table 3:** Inflammation-inducing proteins and associated signaling pathways.

Protein name	Induced inflammatory cytokines	Key signaling pathways	Pathological effects and mechanisms
Tp0751	TNF-α, IL-1β, IL-6 (67)	TLR2/CD14 → MAPK (p38/ERK), NF-κB (11)	Disrupts the blood - brain barrier, facilitating central nervous system invasion (11).
Tp0821	IL-6, IL-1β	TLR2/CD14 → NF-κB (26)	Damages endothelial cells, triggering inflammatory cytokine storms and increasing vascular permeability and local inflammation (26).
TpF1	IL-8, TNF-α, IL-6 (65, 66)	CREB/NF-κB (34)	Induces angiogenesis and macrophage activation (34), increasing cytokine secretion (65).

### 5.1 TpF1

TpF1 is an iron ion-binding oligoferritin with a monomeric molecular weight of approximately 42 kDa. As the core pathogenic antigen of *T. pallidum*, it drives host tissue damage and chronic infection through the multi-target immune regulation mechanism. The core functions of TpF1 are as follows: By activating the cAMP-CREB/NF-κB signaling pathway in endothelial cells (e.g., HUVECs), TpF1 potently induces high expression of IL-8/CXCL8, which subsequently mediates pathological angiogenesis and aberrant endothelial proliferation through an IL-8-dependent (VEGF-independent) mechanism. This pro-angiogenic effect mechanistically relies on IL-8 binding to CXCR1/CXCR2 receptors and has been validated *in vivo*: TpF1 injection significantly promotes angiogenesis concomitant with elevated IL-8 levels in a zebrafish model (34). Concurrently, TpF1 triggered IL-8 acts as a potent neutrophil chemoattractant, driving neutrophil infiltration into the vascular endothelium. This process induces characteristic vascular inflammation (vasculitis) and vessel wall damage, consequently resulting in vascular leakage; furthermore, TpF1 targeted activation of macrophage NLRP3 inflammatory body promotes IL-1β release, and synergistically stimulates T lymphocytes to secrete IL-8, TGF-β and other pro-inflammatory factors, forming persistent inflammatory cascade reaction (65, 66). In addition, TpF1 inhibits host immune clearance ability by inducing regulatory T cell differentiation and activates pathogen-specific T cells to produce pro-inflammatory mediators (66), constructing a pathological network with “pro-inflammatory-immunosuppression” bi-directional imbalance. This multi-dimensional regulatory mechanism not only leads to vascular leakage, central nervous system injury, and other tissue lesions but also provides a key molecular basis for *T. pallidum* to establish chronic infection by evading immune surveillance and maintaining intracellular latency, highlighting its central position in disease progression.

### 5.2 Tp0751

Tp0751 activates MAPK/p38 and NF-κB pathways by binding to TLR2 and CD14 molecules on the surface of monocytic THP-1 cells, induces monocytes THP-1 to release pro-inflammatory factors such as TNF-α, IL-1β, and IL-6 (67). Lu et al. (11) further clarified that Tp0751 induces apoptosis of bEnd3 cells in a concentration-dependent and time-dependent manner, accompanied by caspase-8 activation. It also downregulates tight junction proteins (such as ZO-1 and occludin), and destroys the blood-brain barrier to promote *T. pallidum* invasion into the central nervous system. The dual apoptosis-barrier disruption mechanism exhibits functional complementarity with the pathogenic strategy of *Leptospira* LipL32 protein: LipL32 triggers endothelial cell apoptosis by binding to host fibronectin (68), yet its action is independent of the TLR2 signaling pathway (69), thereby highlighting the pathway-specific selectivity of Tp0751. Meanwhile, Tp0751 significantly promoted IL-6 secretion by bEnd3 cells and exacerbated the inflammatory response by upregulating TNF-α. This multidimensional pathogenic network of “receptor activation–inflammatory cascade–barrier disruption” not only elucidates the core mechanism underlying *T. pallidum* neuroinvasion but also unveils novel avenues for targeted therapeutic interventions.

### 5.3 Tp0821

Tp0821 is an outer membrane lipoprotein of *T. pallidum*, encoding an 749 bp gene containing 249 amino acids, with high immunogenicity and immunoreactivity (70). Tp0821 drives host inflammatory damage and chronic infection through a multi-pathway synergistic mechanism. Its core functions include: Activation of ERK1/2-p38 MAPK/NF-κB signaling axis significantly induced IL-6, IL-8 and IL-1β secretion by monocytes/macrophages. The pro-inflammatory mechanism of Tp0821 shares homology with the NF-κB activation strategy employed by *Helicobacter pylori* CagA protein, yet differs in its mode of action: while CagA relies on a type IV secretion system (T4SS) to invade host cells, Tp0821 directly triggers signal transduction through outer membrane contact (71). Direct cytotoxicity to macrophages, causing LDH leakage and NO release in a dose-dependent manner. This mechanism exhibits functional overlap with Mycobacterium tuberculosis ESAT-6 protein-induced pyroptosis, though Tp0821-induced cytotoxicity primarily involves membrane damage and inflammatory mediator release rather than canonical NLRP3 inflammasome activation (72); Immune escape (late antibody titer decay) is achieved through low adventitial exposure, CD14/TLR2 interaction, and epitope variation; synergistic activation of endothelial ICAM-1/E-selectin and IL-8 increases vascular leakage and neuroinflammatory risk (26). This “inflammation activation-immunosuppression-tissue destruction” multidimensional network not only clarifies the molecular mechanism of syphilis vasculitis and neuropathy but also provides a core strategy for the chronicity of pathogens, highlighting its key value as a diagnostic target.

## 6 Conclusion and perspective

Despite the advancements made in understanding the pathogenic functions of *T. pallidum* proteins, several challenges impede further progress (73). One major obstacle is the bacterium’s intricate growth requirements, which hinder comprehensive analyses of its proteome (74). The demanding nature of these growth conditions presents a barrier to in-depth studies of *T. pallidum* proteins. In recent years, the increasingly severe problem of antimicrobial resistance (AMR) in syphilis treatment has further compounded research challenges — 76.2% of globally circulating SS14 strain isolates harbor the TP0705(A1873G) mutation conferring partial resistance to β-lactam antibiotics (e.g., penicillin G and ceftriaxone) (75), while 99.2% of strains in North America exhibit genotypic resistance to the macrolide azithromycin (76). Moreover, the diverse clinical manifestations of syphilis, combined with individual variations among patients, complicate investigations into the mechanisms underlying the disease (77). The multifaceted roles of *T. pallidum* proteins and their intricate interactions with each other further add layers of complexity to mechanistic studies, making it challenging to unravel the precise pathways involved in disease progression.

To overcome these challenges, upcoming technological advancements such as CRISPR-Cas9-mediated gene editing, Activity-Based Protein Profiling, single-cell sequencing, and metabolomics are poised to play a crucial role. CRISPR-Cas9 technology can create *T. pallidum* strains with specific gene modifications, enabling researchers to conduct more precise evaluations of virulence factors like the adhesin Tp0751 or the metalloprotease Tp0926. Single-cell sequencing and metabolomics approaches hold promise in providing detailed insights into the dynamic interactions between hosts and pathogens, including host-specific metabolic reprogramming during infection (78). Additionally, emerging computational tools—such as Alpha Fold-driven structural predictions and deep learning-based functional annotation—are revolutionizing the decoding of enigmatic proteins like the Tpr family adhesins or the proposed “stealth factor” Tp0435, bridging gaps left by experimental limitations. Collaborative efforts that span disciplines and geographic boundaries are essential to accelerate progress in this field. By fostering interdisciplinary and global initiatives, researchers can collectively address the challenges posed by *T. pallidum* pathogenesis and drive advancements in understanding the roles of its functional proteins.

In conclusion, the functional proteins of *T. pallidum* play a central role in its pathogenicity. Unraveling the specific functions of these proteins not only advances our knowledge of syphilis pathogenesis but also paves the way for the development of innovative diagnostics, vaccines, and therapeutics. Given the escalating threat of drug resistance confronting current first-line therapeutics, the pursuit of proteomics-driven vaccine development and the exploration of novel antibacterial targets have become an urgent imperative. Proteome-wide interaction network modeling, for instance, may uncover novel virulence complexes such as the Tp0136-Tp0326 membrane synergy, while CRISPR-validated studies could clarify the oligomeric pore-forming capacity of Tp0453. Despite the biological complexity of *T. pallidum* and the limitations in laboratory cultivation, the integration of predictive technologies (e.g., comparative genomics-guided domain mapping) with single-cell host-pathogen profiling holds great promise for yielding transformative insights into *T. pallidum* pathogenesis. Notably, breakthroughs have emerged in host-targeted therapeutics: microRNA-based intervention strategies demonstrate therapeutic potential by modulating immune pathways. Specifically, Long et al. demonstrated that miR-223-3p suppresses Tp17-induced pyroptosis through direct targeting of the NLRP3 inflammasome (79). Concurrently, Huang’s team revealed that miR-101-3p downregulates TLR2 expression via direct binding to its 3’ untranslated region (3’UTR), thereby suppressing both transcriptional and translational expression of TLR2 (80). This mechanism subsequently inhibits pro-inflammatory cytokine production (e.g., IL-1β, TNF-α) in Treponema pallidum-stimulated macrophages, thus offering novel avenues for immune pathology containment.

As studies continue to deepen, it is reasonable to anticipate that the synergistic application of predictive modeling and cutting-edge experimentation will not only demystify antigenic variation mechanisms but also catalyze breakthroughs in structure-guided vaccine design and host-directed therapies. Ultimately, enhanced global collaboration and technological convergence may turn the tide against this ancient yet persistently enigmatic pathogen.
